# Impact of and research priorities in early onset epilepsy: An investigation of parental concerns

**DOI:** 10.1016/j.yebeh.2024.109794

**Published:** 2024-07

**Authors:** Natasha Lindsay, Jessica Martin, Dolapo Adegboye, Michael Absoud, Tony Charman, Charlotte Tye

**Affiliations:** a Department of Psychology, Institute of Psychiatry, Psychology & Neuroscience, King’s College London, London, UK; b Department of Children's Neurosciences, Evelina London Children's Healthcare, Guy's and St Thomas' NHS Foundation Trust, London SE1 7EH, UK; c Department of Women and Children's Health, Faculty of Life Sciences and Medicine, School of Life Course Sciences, King's College London, London, UK

**Keywords:** Epilepsy, Development, Parents, Early-Onset, Research Priorities

## Abstract

**[Background and aim]** Early onset epilepsy is a neurological condition with significant developmental consequences, and presents affected children and families with challenges which pervade many aspects of family life. Whilst the concerns of parents and the impact on quality of life is well documented in qualitative research, little emphasis has been placed on the context of ‘early onset’, and the implications of these concerns for research priority setting. We aimed to explore parental perspectives regarding concerns and the impact of early onset epilepsy on the child and family, and to identify priorities for future paediatric epilepsy research.

**[Methods**] The Brain development in Early Epilepsy: Parent Priorities (BEE-PP) project employed a mixed methods approach to collect information on parents’ experience of having a child diagnosed with early onset epilepsy before 36 months old and aged up to 16 years old. Parents completed an online survey (*n* = 15) followed by a focus group (*n* = 5) to explore their main concerns regarding early onset epilepsy, the impact on family life and research priorities.

**[Results]** A thematic analysis of the focus group data generated eight themes related to concerns of parents, the impact on the family and research priorities. The three main concerns identified were the expected trajectory of their child’s development, a lack of seizure control following diagnosis and adverse behavioural side effects of medication. Within family life, early onset epilepsy had an impact on sibling autonomy and psychosocial adaptation, poorer parental wellbeing and restricted social and personal activities. The need for clearer information regarding their child’s developmental trajectory, and managing the side effects of medication and their interactions with behaviour over time were topics of priority for future epilepsy research.

**[Interpretation]** The impact of early onset epilepsy on the family is pervasive and requires the provision of appropriate healthcare service-led support for families to improve quality of life and children’s adjustment to epilepsy. Regular monitoring of the concerns of parents and the impact of the diagnosis would be beneficial for addressing epilepsy-related and psychosocial needs of the wider family throughout their child’s development. Implications for future research priority setting with regards to improved clinician-to-parent information sharing and managing the behavioural side effects of medication are discussed.

## Highlights

• Parents raised profound concerns regarding their child’s early onset epilepsy, including the expected trajectory of their child’s development, an ongoing lack of seizure control and adverse behavioural side effects of medication.
• The perceived impact of childhood epilepsy pervaded several aspects of family life, such as increased sibling autonomy and worsened psychosocial adaptation, deteriorating parental mental wellbeing and greater difficulty in engaging with social activities and personal goals.
• Providing accessible, jargon-free information regarding childhood epilepsy, seizure management and associated developmental trajectories is integral to alleviating parental concern.
• Provision of trauma-informed, patient and family-centered care by healthcare services is needed to improve familial quality of life and children’s adjustment to epilepsy.

## 1 Introduction

Early onset epilepsy is one of the most common, heterogenous neurological disorders of childhood, affecting around 61 in 100,000 individuals per year before the age of 5 [1]. Epidemiological and clinical studies commonly define ‘early onset’ epilepsy as occurring before 3 years of age [2], [3], however this can range up to 5 years old [1]. The increased likelihood of cognitive and behavioural comorbidity, and of early mortality presents early onset epilepsy as a serious medical concern [4]. Infantile spasms, characterised by a sudden bending forward of the body with stiffening of the arms and legs lasting for a few seconds, and focal seizures which occur in a specific part of the brain, are the most common seizure types in infancy [5]. Both infantile spasms and focal seizures have previously been associated with medication treatment-resistance and neurobehavioural difficulties, including intellectual disability, developmental regression and autism [6]. A diagnosis of epilepsy in the first few years of life can have pervasive, and often adverse effects on the child and family, leading to many health and development-related concerns. Past research has indicated a bidirectional relationship between family environment and the child’s adjustment to epilepsy [7] due to practical and emotional support provided by the family. Hence, the familial context is key in supporting the development of a child diagnosed with early onset epilepsy, and may inform the clinical support provided to families [6].

Parents of children with epilepsy are presented with many unique challenges in managing their child’s needs. Qualitative investigations exploring concerns of parents in paediatric epilepsy have identified a lack of epilepsy-related information [8], future of their child with epilepsy [9], side effects of antiseizure medication [10], child's self-esteem and relationships [8], relationships with healthcare providers and epilepsy-related stigma [11], as recurrent sources of worry. Evidence also highlights that parents experience considerable psychosocial, emotional and health-related difficulty following their child’s epilepsy diagnosis, including reduced quality of life and an increased likelihood of developing anxiety and depressive symptoms [12], [13], [14]. Whilst the literature has documented parental fears within various epilepsy cohorts, very little emphasis has been placed on the context of ‘early onset’, and the problems faced by families from a developmental perspective. The wider family context has also received little attention, with the role of siblings often not included in research investigating the impact of having a child with epilepsy [15]. Hence, gaining a holistic understanding of the impact of early onset epilepsy on families may encourage an integrated care approach which takes into account the varied and complex challenges in early life epilepsy.

Addressing parents’ concerns and the impact of early onset epilepsy is fundamental to improving clinical practice, paediatric epilepsy research and patient outcomes [16]. Previous qualitative studies have demonstrated the utility of focus group and interview methodology to gain a detailed and nuanced insight into the familial experience of having a child with epilepsy. Employing a mixed methods approach provides greater objectivity of the reported experiences. Despite this, there is very limited research on the perceived research priorities of parents in the context of childhood epilepsy and early development.

To address these gaps, the objectives of the present research are two-fold: firstly, to document the concerns of parents of children diagnosed with early onset epilepsy (prior to 3 years of age) and its impact on family life, in order to inform the provision of appropriate support for families during a period of complex, rapid neurodevelopmental change. Secondly, we aimed to identify topics which parents feel should be prioritised in future paediatric epilepsy research.

## 2 Methods

### 2.1 Design

Data were collected via the Brain development in Early Epilepsy (BEE) study’s integrated patient and public involvement project, titled ‘Co-production of research priorities for infant epilepsy: the Brain development in Early Epilepsy Parent Priorities (BEE-PP) project’. Parents were invited to take part in an online survey and then a subset of parents who completed the survey consented to join an optional online focus group to further explore their experiences of having a child diagnosed with early onset epilepsy.

All parents gave informed consent to take part in the study. The King’s College London Psychiatry, Nursing and Midwifery Research Ethics Subcommittee provided ethical approval for the study (REC ref: HR/DP-20/21–23531).

### 2.2 Participants

Parents and carers of children aged up to 15 years 11 months old who were diagnosed with epilepsy between 1-month and 36-months old were invited to participate in an online survey. Children were not required to have active epilepsy, defined as having a seizure within the last two years, in order for their parent to participate. Exclusion criteria included infants whose seizures were provoked by acute conditions, such as fevers, infections, trauma, electrolyte disturbances, transient metabolic, and/or endocrine disorders. A total of 15 parents completed the survey. An additional fifteen parents started the survey but did not progress beyond the parent/child demographics section, therefore these data were excluded from analysis. The sociodemographic and clinical characteristics of the participants are described in Table 1.

**Table 1:** Sociodemographic and clinical characteristics of the survey sample.

	N (%)	Median (IQR)^a^
**Parents (*n* = 15)**		
Gender		
Female	11 (73 %)	
Male	4 (26 %)	
Age (years)	−	38
		(35–42.5)
Level of education		
Postgraduate degree or equivalent	5 (33 %)	
Undergraduate degree or professional qualification	7 (46 %)	
A-levels or equivalent	2 (13 %)	
Completed post-16 vocational	1 (6 %)	
course (e.g., apprenticeship)		
Ethnicity		
White – British, Irish, Other	14 (93 %)	
Mixed race – White and Black/Black British	1 (6 %)	
**Children diagnosed with epilepsy**		
Gender		
Female	9 (60 %)	
Male	6 (40 %)	
Age (years)	−	5 years (2.5 – 7 years)
Age at epilepsy diagnosis	−	4 months (3 – 5 months)
Education status		
Secondary school	2 (13 %)	
Primary school	6 (40 %)	
Special provision	1 (6 %)	
Nursery	4 (26 %)	
Not in education	2 (13 %)	
Has sibling(s)	10 (67 %)	
Unknown/missing data	1 (6 %)	
Active epilepsy*^b^*	11 (73 %)	
*Seizure type^c^*		
Infantile spasms	5 (33 %)	
Tonic clonic	4 (26 %)	
Focal aware	8 (53 %)	
Myoclonic	1 (6 %)	
*Epilepsy aetiology*		
Genetic	7 (46 %)	
Structural	7 (46 %)	
Unknown	1 (6 %)	

^*a*^ Interquartile range; *^b^* Seizures occurring within the last two years *^c^*Not mutually exclusive.

Five parents who completed the survey attended the focus group. All participants were female and aged between 35 and 50 years old. Two charity representatives from Young Epilepsy and Epilepsy Research UK also consented to take part in the focus group in order to facilitate a supportive environment and increase information sharing between the epilepsy community and individuals advocating for parents’ needs. However, their contributions to discussions were excluded from the current data analysis to ensure that only parental experiences were captured.

### 2.3 Quantitative measures

An online survey was designed on the platform Qualtrics to collect data on demographic information, epilepsy status, the concerns of parents regarding their child’s epilepsy, the impact of child epilepsy on the family and research priorities (Appendix A). The survey was developed by doctoral students (JM, NL) and the study principal investigator (CT). As part of the screening checks, IP addresses (which indicate the location of the respondent) were recorded on Qualtrics for all survey respondents. Locations were checked by the research team to ensure responses were from parents based in the UK and not duplications.

#### 2.3.1 Demographics

Socio-economic, age and gender information were collected for parents and their child (and siblings if applicable).

#### 2.3.2 Parental concerns, family impact and priorities for paediatric epilepsy research

Existing literature published from 2000 onwards was collated by the co-investigator (JM) to inform the online survey questions. A systematic literature review was not undertaken, however the topics searched included (1) the population of interest (i.e., infants and children with early onset epilepsy, parents and siblings of children with epilepsy), (2) the perspectives of interest in the context of epilepsy (i.e., parental concerns and priorities), (3) the impact of early onset epilepsy on the affected child, family unit and siblings. Several standardised questionnaires also informed the online survey. Bespoke questions about parent’s concerns were adapted from the Core Outcome Set (COS) for childhood epilepsies by Crudgington et al. (2019) [35]. In this study, consensus‐based methods were used to rate the importance of different outcomes in rolandic epilepsy using two surveys and a meeting that included young people with epilepsy, parents, and various professionals. Questions regarding the impact of childhood epilepsy were informed by several standardised scales including the Impact of Paediatric Epilepsy (11 items; Camfield, Breau, & Camfield, 2001) [36], Impact on Family Scale (15 items; Stein & Jessop, 2003) [37], Paediatric Quality of Life Inventory-Family Impact Module (36 items; Varni et al., 2004) [38] and a subset of items taken from Kroner et al.’s (2018) bespoke questionnaire [28] which assessed parental perspectives of the impact of epilepsy on siblings of children with epilepsy. Bespoke questions regarding parents’ priorities for future research were informed by the impact and concerns sections of the survey, as well as a report by Berg et al. (2013) [16] which explored priorities in paediatric epilepsy research.

Various topics of parental concern regarding their child’s epilepsy were measured using a 5-point Likert scale ranging from ‘Not at all concerned’ to ‘Extremely concerned’. Topics of priority for paediatric epilepsy research were also assessed using a Likert scale ranging from ‘Not at all important’ to ‘Extremely important’. Open text boxes were provided to allow parents to report concerns or research priorities not listed within the bespoke question list.

Two epilepsy charity representatives from Young Epilepsy and Epilepsy Research UK and a patient representative reviewed the survey to ensure the language and scope of the questions were sensitively worded for the epilepsy community and achieved the goals of the study. Additional questions regarding the child with epilepsy’s educational placement and whether the parent accessed mental health services were also added in response to reviewer feedback. The online survey data was analysed independently of the qualitative data, however some of the results from the survey informed the focus group topic guide.

### 2.4 Qualitative measure

#### 2.4.1 Focus group

A topic guide was developed by a doctoral student (NL), a postdoctoral researcher (DA), the study principal investigator (CT) and a clinical psychologist (TC). Topics covered during the focus group (Table S1) included parents’ perspectives on the value of co-production, the impact of their child’s epilepsy on the family, parent concerns and priorities for future epilepsy research. The questions were developed based on responses to the online survey, for example topics which were not rated as an impact or priority by any parents were not included in the topic guide questions or prompts. Feedback from patient and charity representatives regarding language appropriateness and inclusion of positive impacts also informed the topic guide questions.

The focus group was cofacilitated by two researchers, audio recorded and transcribed in real time via Microsoft Teams. The transcripts were analysed and coded by two doctoral students (NL, DA) and reviewed for discrepancies.

### 2.5 Procedure

Participants were invited to complete an online survey which was distributed electronically on social media platforms, the study website (https://www.beestudy.co.uk) and by emailing UK epilepsy charities. A project information sheet was provided alongside the survey URL link which described the project aims, data collection activities, participant criteria and data usage. During survey completion, an item prompted parents to indicate if they consented to being contacted about taking part in an audio-recorded focus group to further explore their survey responses. Of the parents who completed the survey, 13 consented to being contacted via email to learn more about the focus group. Emails were sent to this subset of parents to ask whether they would like to take part in a 2-hour online focus group hosted by the research team which aimed to explore the impact of their child’s epilepsy and what future research should focus on. Eight parents did not respond or declined to take part, and 5 parents consented to take part and attended the focus group. All parents were offered a £50 reimbursement for their participation. Following the focus group, participants were asked to review additional outputs generated by the project, including a parent-voiced video animation of priorities for research in early-onset epilepsy and an infographic which summarised key findings from the survey.

### 2.6 Data analysis

#### 2.6.1 Quantitative – Online survey

Descriptive analyses were conducted to generate frequencies for the socio-demographic profile of the parents who completed the online survey. Parents’ primary concerns and research priorities regarding early-onset epilepsy were calculated as the proportion of parents who rated the topic as “Extremely/very concerning” or “Extremely/very important”. The sample was split by epilepsy aetiology to examine differences in parents’ concerns and research priorities based on the cause of their child’s epilepsy. Open text data were reported as a summary of the main topics discussed.

#### 2.6.2 Qualitative – Focus group

Thematic analysis was used to analyse the data using NVivo software. In line with guidance on conducting thematic analysis described in the work of Braun and Clarke [17], the data were analysed using an inductive, semantic approach to generate themes.

In keeping with Braun and Clarke’s six phases of thematic analysis, the data were first transcribed and then imported into NVivo for proofreading and re-reading to generate initial ideas that were pertinent to the text. In phase two, the primary data coder (NL) coded features of the text line by line, which were relevant to either a parental concern, an impact on the family or a research priority. In stage three, the codes and individual quotes were collated into potential themes which were associated with one or more of the three categories (a concern, an impact or a research priority). In stage four, NL conceptualized the themes and subthemes to ensure they reflected the full dataset and extracted codes. Within this stage, a thematic map was created to visually represent the relationships between themes and subthemes. Stage five involved further refining each theme by checking that the quotes and codes represented the ‘concept’ of the theme and fit a narrative of parent perspectives based on the full dataset. In the final stage, the write up of the findings involved describing the themes in detail with direct extracts from the transcript to support this. A second coder (DA) completed the above phases with 50 % of the transcript, and discussed the results of the themes with NL to ensure consistency of coding.

## 3 Results - Online survey

### 3.1 Parent-reported concerns

**Fig. 1:**
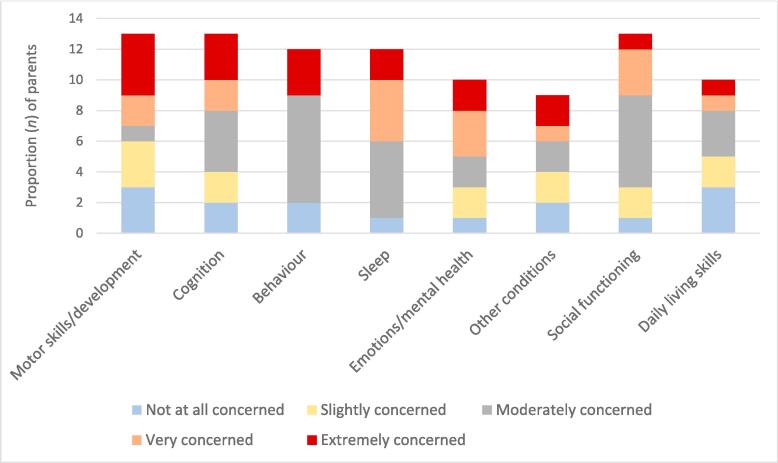
Ranked concerns of parents of children diagnosed with early onset epilepsy.

A summary of the open-text responses from parents (*n* = 8) indicated that concerns for their child included sleep difficulty, in particular regular periods of disrupted sleep or insomnia at night due to increased seizure activity or antiseizure medication use. Other concerns reported were fearing for their child’s future since they are showing developmental delay in early childhood, as well as worries about how their child will develop given their delayed language and communication skills. The quantitative results indicated that half of all parents ranked their child’s sleep as “Very” or “Extremely concerning”, and 46 % of parents ranked motor skills and development as “Very” or “Extremely concerning” (Fig. 1).

**Fig. 2:**
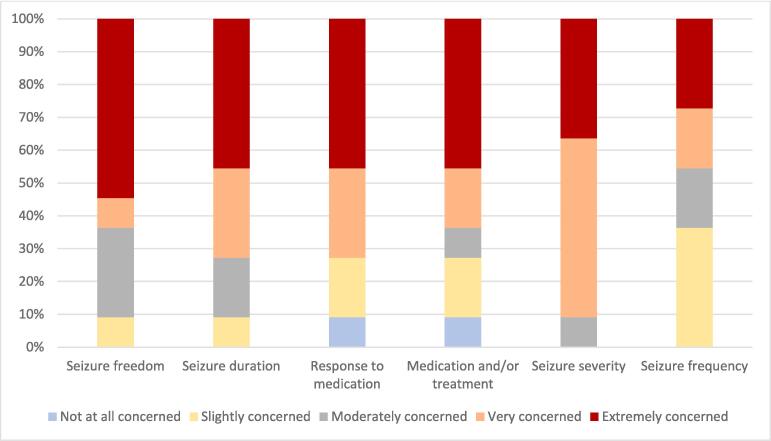
Epilepsy-related concerns of parents of children diagnosed with early onset epilepsy.

Regarding epilepsy-specific concerns, 90 % of parents rated seizure severity as “Extremely” or “Very concerning”, whilst 72 % of parents rated seizure duration and response to medication as “Extremely” or “Very concerning” (Fig. 2).

### 3.2 Concerns by aetiology

Parents of children diagnosed with epilepsy due to a genetic cause (n = 7) ranked cognitive and motor skills as the most concerning areas of their child’s development (86 % of parents rating as “Extremely” or “Very Concerning”), whereas 86 % parents of children with no known cause of their epilepsy (n = 7) ranked sleep and emotional wellbeing as their highest concern.

### 3.3 Parent-reported research priorities

A summary of the open text responses (n = 2) indicated that parents perceive the healthcare and community support received following the epilepsy diagnosis as “inadequate”. One parent highlighted that research should focus on documenting the complex challenges that parents take on in order to inform appropriate support for caregivers. Another parent commented on the “huge need for medications that do not cause or exacerbate emotional and behavioural issues”.

**Fig. 3:**
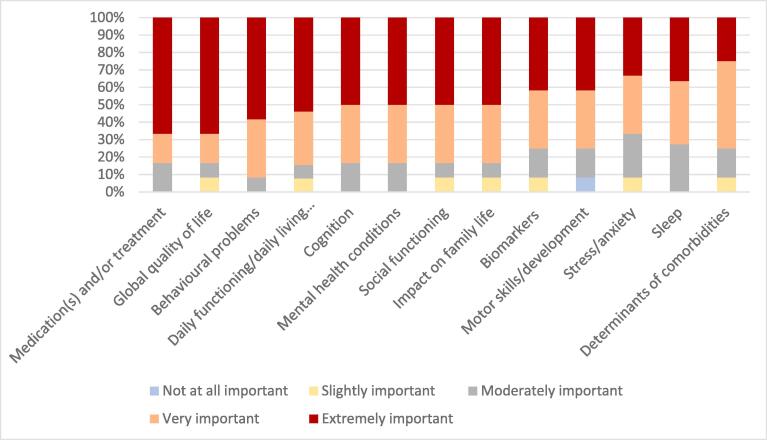
Ranked research priorities for parents of children diagnosed with early onset epilepsy.

The highest ranking research priorities in the full sample were behavioural problems (91 % of parents rating as “Extremely” or “Very Important”) and daily living skills (84 % of parents rating as “Extremely” or “Very Important”) (Fig. 3).

### 3.4 Priorities by aetiology

Parents of children diagnosed with epilepsy due to a genetic cause (n = 7) ranked medications/treatment and biomarkers of developmental outcomes as the most important topics for future research (86 % of parents rating as “Extremely” or “Very Important”). Parents of children with no known cause of their epilepsy (n = 7) ranked medications/treatments and determinants of co-occurring conditions as highest ranking priorities for future research (71 % of parents rating as “Extremely” or “Very Important”).

## 4 Qualitative results: Thematic analysis of focus group

(Fig. 4)

**Fig. 4:**
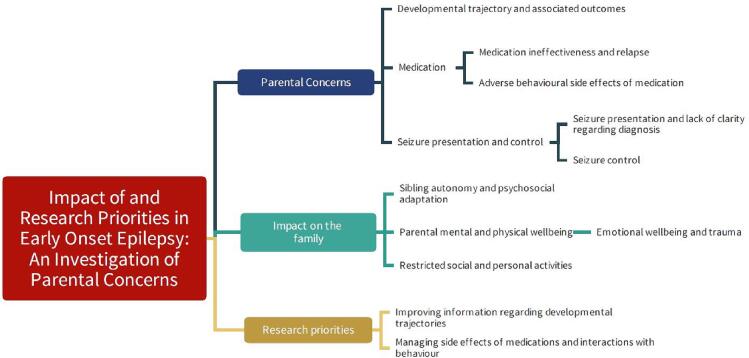
Thematic map of focus group findings.

**Parental concerns and research priority themes.**

### 4.1 Developmental trajectory and associated outcomes

A prevalent concern emerging from caregiver discussions was a paucity of clear, jargon-free information regarding the expected developmental trajectory and outcomes for children with early onset epilepsy. The primary source of this information derived from healthcare professionals involved in their child’s care (e.g., neurologists or epilepsy nurses) and research study literature accessed independently by parents. Parents acknowledged that “incredibly varied and complicated” outcomes in early onset epilepsy contributes to the challenge in predicting a trajectory of development following diagnosis. One parent stated that “sometimes doctors can rely very heavily on a sort of textbook expected evolution of how things will go, whereas actually we know that in reality, epilepsy is incredibly varied”. A lack of clear information regarding development caused parents to worry about what to expect for their child’s psychosocial and intellectual development, including academic [“he's not getting things in school in the way that he should”], social [“he's also not forming friends in the way that other kids are”] and physical [“And then he just stopped growing, and I don't know what's going on”] concerns.

Parents reported that medical information regarding intellectual, social and physical development and associated outcomes was generally lacking in detail, “overly complex or basic” or not considering the context/nuance of each families’ circumstances, which further contributed to parents’ worries. Ultimately, the medical prognosis given by expert clinicians and sought out in the literature “did not provide a full picture” for most parents and was considered a priority for clinical services to improve and for research efforts to focus on.

### 4.2 Medication

#### 4.2.1 Medication ineffectiveness and relapse

This theme refers to parental concerns regarding medication effectiveness and the unpredictability of seizure relapse following a period of medication effectiveness. Changes in antiseizure medication introduced by the primary clinician often heightened parents’ fears because there was little indication as to whether it would improve seizure presentation or severity.

Parents experienced fluctuating feelings of hopefulness and worry, in which medication effectiveness (indicated by reduction or cessation of seizures) was followed by stressful and unpredictable periods of ineffectiveness (indicated by seizure relapse or continuation/increased severity of seizures). The recurrent feelings of hopelessness were expressed as a lack of faith in medication development as a viable solution for reducing or eliminating seizures. The cause of sudden medication ineffectiveness was also unclear to parents which became a further source of worry, with medication resistance cited as a possible explanation. Even where relapse has not occurred following successful medication use, concerns remained about whether seizure freedom will remain for their child and for how long. One parent stated “even though our son’s epilepsy is more or less controlled now, we still have this constant worry and anxiety about whether this will stay this way, or whether he will have a seizure the next day”. These anxieties were experienced over a number of years post-cessation of seizures, with many parents relying on “hope” to maintain medication freedom, and considering those that do as “lucky”.

Medication changes (as a result of inability to reduce seizure activity) were viewed as a “horrendous”, or “scary” time for some parents due to the inability to know whether their child would respond well i.e., present with less seizures. This uncertainty led to a lack of faith in any one particular medicine to ‘cure’ the epilepsy.

#### 4.2.2 Adverse behavioural side effects of medication

The side effects of antiseizure medication, namely sleep and behavioural problems, were a prevalent theme of concern and a research priority for all parents. Specific medications and increasing the dosage of medication appeared to trigger problematic side effects. Parents expressed that “some of the medication that she's been on just has given her complete insomnia” and “our previous consultant kept increasing his dose of carbamazepine until we could basically hardly keep him awake in the morning”. Children’s sleep difficulties, in particular night-time insomnia and daytime sleepiness, were deemed a detriment to three parents’ wellbeing. Children’s sleep problems were discussed as a key contributor to parents’ disrupted sleep, physical and mental exhaustion − one parent describing that “it makes you go crazy” and “I have fantasies about lying down and just sleeping”. Parents also reported that medical professionals involved in their child’s care showed a lack of regard for sleep problems, due to value being placed on the potential therapeutic benefits of the antiseizure medication over the side effects which impact parent and family wellbeing. Other behavioural side effects, including medication-induced temper tantrums and loss of impulse control were described as severely concerning for one parent, due to the threat to their child and other’s safety.

Although medication effectiveness was a top research priority for parents, they emphasised the importance of funding research which could: (1) find a “better balance” between seizure control using antiseizure medication and reducing the debilitating side effects of antiseizure medication, and (2) describe the impact of medication use on developmental ability and behaviour in a clear, digestible way for families’ which highlights what to expect for their child’s development.

### 4.3 Seizure presentation and control

#### 4.3.1 Seizure presentation and lack of clarity regarding diagnosis

Nearly all parents referred to pervasive worries about the presentation and severity of their child’s seizures. Seizures longer in duration, higher in frequency and which required hospitalisation were of particular concern due to the implications of the illness on overall health and likelihood of mortality. Parents also voiced frustration about their child’s epilepsy diagnosis. These concerns were related to unclear information provided by healthcare professionals regarding the clinical difference between seizure types, as well as difficulty in confirming the diagnosis based on the seizure presentation in such a young infant. A lack of clarity led some parents to seek answers by engaging in their own independent research regarding seizure diagnostic criteria.

#### 4.3.2 Seizure control

An ongoing lack of seizure control was a concern referenced by all parents at some stage throughout discussions. Fears of ongoing seizure presence or seizure relapse reflected medication ineffectiveness (as discussed in subtheme 4.2.1) or sleep-induced seizures. In some cases, there was no identifiable cause for the seizures. In these cases, parents reported intense worrying about how to reduce seizure activity without a clear trigger (such as environmental and/or neurobiological risk factors) identified by medical professionals. The uncontrolled and unpredictable nature of the seizures has meant that for most parents their child’s health has been consistently unstable over a number of years.

**Family impact themes**

### 4.4 Sibling autonomy and psychosocial adaptation

Parents acknowledged and expressed concern regarding the impact of siblings’ exposure to their child’s seizures. Worries often stemmed from the belief that the sibling would find difficulty managing their own needs due to a reduced amount of attention and support received from parents, as a result of increased attention paid to the needs of the child with epilepsy. Parents reported a lack of capacity to attend to a siblings needs which led to siblings prematurely taking on a considerable level of independence to ‘get on with’ daily tasks, such as homework or games, by themselves. Two parents sought professional help in the form of counselling to support siblings. One parent observed strategies used by siblings to cope with the challenges, including avoidance of seizure episodes occurring in the family home.

The impact on sibling wellbeing and behaviour is not just limited to home life, but also affects schooling. Two parents reflected on the mental ‘pressure and toll’ on siblings, which has led to inappropriate responses to conflict at school, intensified emotional responses, behaviourally “acting out” and issues maintaining friendships.

### 4.5 Parental mental and physical wellbeing

Parents mental wellbeing and identity were all significant areas of impact in the context of their child’s epilepsy. Nearly all parents experienced worsened mental and emotional wellbeing and/or trauma in the context of managing the day-to-day care of their child, specifically during periods of recurrent and severe seizures.

#### 4.5.1 Emotional wellbeing and trauma

Parental emotional wellbeing difficulties were experienced following frequent exposure to and management of their child’s seizure episodes and challenging behaviours, which could range from less severe to life threatening and requiring urgent medical intervention. One parent stated “I can't tell you how many times we've been in hospital because it's so many. It's ridiculous. He's had an hour, hour and a half seizures, hour and a half in status epilepticus. That's nuts. You know that, that is crazy. But you know it's on me and I think that's the thing. The very heavy burden that you feel. Uhm, is impactful is really, really impactful.” Parents emphasised that the impact of providing constant care for a child with epilepsy had led to a sense of trauma, which pervades all aspects of family life. By playing the critical role of primary advocator and expert-by-experience for their child’s complex needs, there was a significant mental toll experienced over time. There was also a perceived lack of understanding from others concerning the level of trauma that the family experience, because the challenges they face are epilepsy-specific and can range in severity.

Poorer mental and emotional wellbeing was expressed in many ways among the group. Two parents referred to an “underlying anxiety” or a “low level alertness” which is constantly present and waits in anticipation of the next seizure. Two other parents resonated with feelings of “helplessness”, “emotionless” and inability “to cope with the stress” of their child’s health related issues. To manage these feelings, parents described coping mechanisms including the avoidance of epilepsy-related medical information regarding development (for fear of knowing more about future outcomes) and remaining on ‘autopilot’, so as to not feel the heavy emotional impact. Although mental strain took precedence over the physical impact, two parents referred to “chronic exhaustion” and “terribly tense shoulders” as a result of being the primary care provider for their child’s needs.

#### 4.5.2 Restricted social and personal activities

There was a consensus among parents that making future social and personal plans had become more challenging as a result of their child’s epilepsy. Planning and engaging in social or recreational events for the family were deemed “not worth it” and could even result in added stress for the family due to the unpredictability of seizures. There was a level of convenience expressed in managing their child’s epilepsy at home, and hence the pre-planning required for social outings were considered a “hassle”. This was not only limited to larger-scale family plans such as holidays, but also daily/weekly activities such as going to the shop or arranging family dinners outside of the home.

Parents personal goals and needs were also affected by the long-term management of their child’s epilepsy. Four parents described the constant prioritisation of their child with epilepsy over their own needs, which has impacted many aspects of their personal life. The inability to arrange basic health checks, to exercise, to travel, to pursue certain job opportunities and to remain in permanent employment were some of the examples given, with the main reason cited as difficulty with time management. The discussions portrayed a widespread impact of epilepsy care on parents’ personal goals and freedom to make decisions which could improve their physical health and wellbeing.

## 5 Discussion

The present study contributes to our understanding of the impact of having a child with early onset epilepsy, and identified priorities for future paediatric epilepsy research. The themes derived from parents’ concerns were the expected trajectory of their child’s development, a lack of seizure control following diagnosis and adverse behavioural side effects of medication. The perceived impact of childhood epilepsy pervaded many aspects of family life, in particular poorer sibling and parental wellbeing, as well as the inability to engage with social, leisure and personal activities. The available information regarding developmental trajectory, and how medication interacts with later behaviours was perceived as insufficient and unclear, and hence considered a priority for future epilepsy research.

### 5.1 Parental concerns

Despite differences in seizure status and severity among the focus group participants, all parents expressed persistent worry regarding the presentation and control of their child’s seizures. Investigations exploring families’ experiences of having a child with epilepsy have identified similar topics of concern, including poor seizure control [9], [18], the unexpectedness of seizures [19] and seizure activity worsening [20]. Such anxieties were present regardless of their child’s age or seizure status, which reflects the need for ongoing support in managing seizures throughout development. In our data, concerns regarding seizure relapse were most common among parents whose children had medication-resistant epilepsy, and these parents reported a lack of faith in long term medication effectiveness – a common intervention for early onset epilepsy. Hence, ensuring clear clinician-to-parent information sharing regarding medication changes and checking parents understanding should be prioritised to alleviate worries and support children’s adjustment to epilepsy.

Delayed cognitive and motor development were two major areas of concern for parents of children diagnosed with epilepsy due to a genetic cause. Aetiology of epilepsy is among many factors that can have a considerable impact on intellectual outcomes [4]. An increased likelihood of impaired cognitive development has been found in individuals with an identified aetiology (e.g., genetic or structural) as opposed to idiopathic causes [21], which reflects the concerns of parents in our study. Such evidence supports early diagnostic and genetic testing in epilepsy which could inform therapy development for improving cognitive outcomes.

Parents’ concern regarding the adverse behavioural side effects of medication was a recurring theme in the focus group discussions and survey. Several studies highlight parents’ views on the negative impact of antiseizure medication on behaviour, citing increased tiredness in the child [22], [23], mood-related behaviour problems [24], and a preference for medication changes when the side effects were too impactful [25]. Parents reported that clinicians’ decision to focus on medication effectiveness over behaviour problems further exacerbated their concerns, indicating that recognition of the full range of difficulties in childhood epilepsy is fundamental to comprehensive epilepsy care.

### 5.2 Family impact

There is consistent evidence indicating that caring for a child with epilepsy contributes to worsened family quality of life and mental health [14]. In our study, parents’ inability to engage in social activities and personal goals was a byproduct of prioritizing their child's epilepsy-related needs. Similar outcomes have previously been reported, whereby parents of children with epilepsy experience increased isolation due to avoidance of social activities, limiting travel and leaving employment [26]. A review study concluded that reduced quality of life in parents of children with epilepsy was associated with greater anxiety and depressive symptoms and reduced quality of life in the affected child, but not with seizure control [27]. Our focus group discussions however, suggested seizure presence was a major contributor of worsened parental mental wellbeing. This may be explained by the nature of ‘early onset’, which requires full caregiver management of seizures in early infancy. It is likely then, that a combination of epilepsy-related factors and family environment/psychosocial factors are integral for improving family quality of life and wellbeing.

Our findings identified varied coping strategies employed by parents and siblings, including avoidance of seizure episodes and epilepsy-related information, and actively seeking out epilepsy-related information. Employing effective coping strategies suggests a sense of resilience in families, which has been noted as a positive outcome in several studies of siblings and parents of children with epilepsy [28], [29]. Given that previous research has observed a bidirectional relationship between parental wellbeing and children’s adjustment to epilepsy [27], our findings have important implications for the provision of appropriate support for parents to ensure their capacity to manage their child’s needs are met. Research has supported the development of patient and family-centered care models for children with chronic health problems, and specifically epilepsy, in order to improve quality of life for families and enhance the quality of communication between parents and healthcare professionals [30], [31]. Hence, prioritising trauma-informed care may be key for supporting families’ wellbeing and adjustment to epilepsy.

### 5.3 Research priorities

The research priorities identified in focus group discussions are consistent with those derived from the European Forum on Epilepsy Research, which highlighted the development of biomarkers and identifying factors that lead to cognitive impairment and behavioural concerns as key areas of priority [11]. Further evidence from a study of parents of newborns with epilepsy concluded that a high priority research topic for parents was the adverse effects that antiseizure medication may have on neurodevelopment [32]. Focus group discussions echoed these findings, as parents expressed the need for research efforts to focus on comprehensive, jargon-free information regarding their child’s developmental trajectory, as well as understanding the behavioural side effects of medication. In concordance with these views, a systematic review investigating the information needs of parents of children with early onset epilepsy concluded that parents need understandable information about early onset epilepsy and associated comorbidities throughout their child’s lifespan [10]. The desire for more information regarding psychosocial and intellectual outcomes in epilepsy identified in this and previous studies indicates the importance of developing evidence-based interventions for the developmental and learning needs of young children with epilepsy. In support, a recent mixed methods investigation concluded that providing epilepsy-related psychoeducational interventions to primary caregivers of children with epilepsy further enables parents to support their child’s psychosocial needs [33].

### 5.4 Limitations

The small sample of parents who completed the online survey and took part in the focus group limits the generalizability of our data and contributed to a lack of representativeness in the sample, particularly with regards to ethnic and socio-economic characteristics of parents. We acknowledge that the findings from this homogenous group may limit our understanding of the diverse range of experiences inherent in investigating parental perspectives on early onset epilepsy. Alternatives to first-line antiseizure medications, such as vagus nerve stimulator therapy, the ketogenic diet and epilepsy surgery were also not captured in our data despite a wide variation in epilepsy status and severity among the focus group participants. As a result, differences in parent perspectives may emerge based on access to other treatment modalities. A further limitation is reliance on accurate parent recall from the point of diagnosis and throughout development which may skew the accounts given. The sample also included parents of children with both active seizures (of varying severity), and those who are seizure free, which limits our understanding of the influence of epilepsy status and type. Increased feelings of social isolation and misinformed parental perceptions of epilepsy has previously been reported in children with higher severity of seizures compared to low severity groups [34], highlighting the importance of seizure status in the impact on families.

### 5.5 Conclusion

Gaining an insight into the concerns of parents and the impact of epilepsy on the family is a valuable qualitative source of information for service providers and researchers involved in paediatric epilepsy care. The data suggests that the development and provision of appropriate support for families is key to improving family wellbeing and child development, in particular by providing accessible epilepsy-related information regarding diagnosis, seizure management and future outcomes which is specific to the families’ needs. As identified by parents, research efforts therefore may benefit from focusing on the family-clinician relationship, with regards to information-sharing and continual monitoring of concerns to ensure families and children diagnosed with early life epilepsy are fully supported.

## CRediT authorship contribution statement

**Natasha Lindsay:** Writing – review & editing, Writing – original draft, Visualization, Validation, Resources, Project administration, Methodology, Investigation, Funding acquisition, Formal analysis, Data curation, Conceptualization. **Jessica Martin:** Writing – review & editing, Resources, Project administration, Methodology, Investigation, Funding acquisition, Data curation, Conceptualization. **Dolapo Adegboye:** Writing – review & editing, Project administration, Methodology, Investigation, Formal analysis. **Michael Absoud:** Writing – review & editing. **Tony Charman:** Writing – review & editing, Supervision, Methodology, Investigation, Conceptualization. **Charlotte Tye:** Writing – review & editing, Supervision, Resources, Project administration, Methodology, Investigation, Funding acquisition, Data curation, Conceptualization.

## Declaration of competing interest

Dr. Charman has acted as a paid consultant to F. Hoffmann-La Roche Ltd. and Servier; and has received royalties from Sage Publications and Guilford Publications.
