# Supplementary material for: Impact of and research priorities in early onset epilepsy: An investigation of parental concerns
Source: Epilepsy Behav. 2024 Jul;156:109794. doi: 10.1016/j.yebeh.2024.109794 (PMC11790341; doi:10.1016/j.yebeh.2024.109794)
Supplement: Supplementary data 1 [file mmc1.docx]

**Supplementary Material**

**Contents**

Table S1. Focus group topic guide questions

Appendix A. Online survey questions

**Table S1. Focus group topic guide questions**

| **Value of co-production** |
| --- |
| Why are collaborative research projects, involving parents or charity representatives such as yourselves, important to you? |
| Why do you think it's important for you or your child to play an active role in epilepsy research? |
| What motivates you to take part in collaborative activities such as this focus group, and share your experiences of having a child with epilepsy? |
| In your experience, have there been enough opportunities to be involved in discussions aimed at shaping future epilepsy research? |
|  |
| **Impact of child’s epilepsy** |
| Could you describe how has your child's epilepsy impacted you and/or your family? |
| How has the condition affected your daily living?  *Prompt*: for example ‘socialness’ as a family, attending family events, holidays or gatherings, or planning for the future? |
| How have these challenges changed from when your child was first diagnosed to later years of their development? |
| What effect has your child’s epilepsy had on your emotional wellbeing, or mental health? And your physical wellbeing? |
| What have you found helpful in dealing with these challenges? |
| What have you found most helpful for yourself, your family and child in managing epilepsy? Could you describe the positives that may have come from your child’s diagnosis, for example closeness as a family? |
| Which services or interventions, if needed, have been the most useful? And what was your experience of gaining access to this support? |
|  |
| **Concerns and research priorities** |
| What is your biggest concern regarding your child’s epilepsy? |
| How do your concerns differ if you consider when your child was first diagnosed to now? |
| What area/topic related to early development and behaviour in epilepsy should be given a spotlight in research, if any, and why?  *Prompt:* Why do you think XX area should be prioritized for future research? |
| What more can be done in research to address the concerns you have or the impact of your child's epilepsy? |
| Are there any other concerns about your child’s epilepsy not discussed today that you would like to speak about? |

**Appendix A.**

**Online survey questions**

**Demographics**

This section of the survey asks about you and your family’s demographics, such as your ethnicity and household income. Responses provided in this section will be used to understand more about our respondents’ background. Other questions ask about your child’s medical diagnosis and these responses will be used to understand the medical impact and your concerns surrounding early epilepsy.

**About you**

1. Please enter the first half of your postcode (e.g., SE5).
2. Free text
3. How old are you? (in years)
4. Free text
5. What is your gender?
6. Male
7. Female
8. Other
9. I’d prefer not to say
10. What is your ethnicity?
11. Asian/British – Indian, Pakistani, Bangladeshi, other
12. Black/Black British – Caribbean, African, other
13. Mixed race – White and Black/Black British
14. Mixed race – other
15. White – British, Irish, other
16. Chinese/Chinese British
17. Middle Eastern/Middle Eastern British – Arab, Turkish, other
18. Other ethnic group
19. I’d prefer not to say
20. What is your highest educational qualification?
21. No qualifications
22. GCSE or equivalent (at school until aged 16)
23. A-levels or equivalent (at school until aged 18)
24. Completed post-16 vocational course (e.g., apprenticeship)
25. Undergraduate degree or professional qualification
26. Postgraduate degree or equivalent
27. I’d prefer not to say
28. What is your total household income (pre-tax)?
29. <£20 000
30. £20 000 - £29 999
31. £30 000 - £39 999
32. £40 000 - £59 999
33. £60 000 - £79 999
34. £80 000 - £99 999
35. £100 000 - £149 999
36. > £149 999
37. I’d prefer not to say
38. Are you a single parent household?
39. Yes
40. No
41. I’d prefer not to say
42. We understand that an early diagnosis of epilepsy can have a great impact, and this may be magnified for parents with additional difficulties. We understand this is a highly sensitive topic, and would be really appreciative of you telling us if you have a diagnosis of any of the following (you may select multiple responses):
43. I’d prefer not to say
44. Clinically diagnosed depression
45. Clinically diagnosed anxiety
46. Attention deficit hyperactivity disorder (ADHD)
47. Autism Spectrum Disorder (ASD)
48. Another clinically diagnosed mental health condition
49. Epilepsy
50. Other (add details)

**About child with epilepsy**

1. What is your child’s date of birth?
2. Day <drop down>
3. Month <drop down>
4. Year <drop down>
5. What is your child’s gender?
6. Male
7. Female
8. Other
9. I’d prefer not to say
10. What is your relationship to your child?
11. Parent
12. Stepparent
13. Grandparent
14. Other
15. I’d prefer not to say
16. What is your child’s ethnicity?
17. Asian/British – Indian, Pakistani, Bangladeshi, other
18. Black/Black British – Caribbean, African, other
19. Mixed race – White and Black/Black British
20. Mixed race – other
21. White – British, Irish, other
22. Chinese/Chinese British
23. Middle Eastern/Middle Eastern British – Arab, Turkish, other
24. Other ethnic group
25. I’d prefer not to say

Q: Where is your child usually educated?

a. Nursery

b. Daycare centre (e.g., a playgroup)

c. Childminder/childcarer

d. Primary school

e. Special provision school

Secondary school

f. Home educated

g. Not in education

h. Other

[only display following Q if child is in education]

Q: Which best describes your child’s place of education?

a. Mainstream state schooling 
b. Private (independent) schooling

c. Special educational needs and/or disabilities schooling

d. Other, please describe: [free text]

1. What type of seizures does your child experience?
2. Free text
3. Does your child have any specific diagnoses?
4. Free text
5. How old was your child when they were diagnosed with epilepsy?
6. Did your child have any seizures or convulsions in the first month of life?
7. Yes
8. No
9. Not sure
10. I’d prefer not to say
11. After the first month of life, did your child ever have a seizure with a fever (sometimes called febrile convulsions)?
12. Yes
13. No
14. Not sure
15. I’d prefer not to say
16. Before the diagnosis of epilepsy, did your child ever have any other seizures that were caused by an acute event such as a head injury concussion or low blood sugar?
17. Yes
18. No
19. Not sure
20. I’d prefer not to say
21. The Early Childhood Epilepsy Severity Scale (6 items; Humphrey et al., 2008).
22. Please rate how worried you feel about the following: (Not at all concerned, Slightly concerned, Moderately concerned, Very concerned, Extremely concerned, N/A)
23. Seizure frequency (How often your child’s seizures occur)
24. Seizure duration (How long your child’s seizures last)
25. Seizure severity (How bad your child’s seizures are in terms of the effect on them during and after a seizure e.g., injuries and confusion)
26. Seizure freedom (Whether your child will become seizure free)
27. Medication(s) and/or treatment (The number of or type of medications and/or treatment)
28. Response to medications and/or treatment (How your child is responding to their mediation(s) and/or treatment).
29. Please describe your concerns about the following: (display if rated concern from Moderately concerned - Extremely concerned on above Q)
30. Seizure frequency (How often your child’s seizures occur)
31. Seizure duration (How long your child’s seizures last)
32. Seizure severity (How bad your child’s seizures are in terms of the effect on then during and after a seizure e.g., injuries and confusion)
33. Seizure freedom (Whether your child will become seizure free)
34. Medication(s) and treatment
35. Response to medication(s) and/or treatment
36. If no to question about medications, do you think your child would benefit from taking any medicines (if appropriate)?
37. Yes (please add details)
38. No
39. I am not sure
40. I’d prefer not to say
41. Has your child been hospitalised/received any medical treatment in hospital that worries you?
42. Yes (please add details)
43. No
44. I am not sure
45. I’d prefer not to say
46. We understand that an early diagnosis of epilepsy can have a great impact, and this may be magnified for children with additional difficulties. We understand this is a highly sensitive topic, and would be really appreciative of you telling us if your child has a diagnosis of any of the following (you may select multiple responses):
47. I’d prefer not to say
48. Autism Spectrum Disorder (ASD)
49. Attention Deficit Hyperactivity Disorder (ADHD)
50. Developmental Coordination Disorder (DCD)/Dyspraxia
51. Conduct Disorder/Oppositional Defiant Disorder
52. Anxiety Disorder
53. Mood Disorder
54. Tourette’s Disorder/Tic Disorder
55. Specific learning difficulties
56. General learning disability/Intellectual Disability/Developmental Delay
57. Does your child with epilepsy have any siblings? Your response will filter suitable questions.
58. Yes
59. No
